# Nanosecond pulse electric field treatment initiates mitochondrial apoptosis pathway by inducing mitochondrial morphological changes in myocardial cells

**DOI:** 10.1007/s10840-024-01828-5

**Published:** 2024-08-02

**Authors:** Aqing Fan, Gengliang Liu, Xiaodong Wu

**Affiliations:** 1 University of Science and Technology of China, Hefei, 230000 China; 2 Suzhou Institute of Biomedical Engineering Technology, Chinese Academy of Sciences, No. 88 Keling Road, Huqiu District, Suzhou City, 215163 Jiangsu Province China

**Keywords:** Nanosecond pulse electric fields, Myocardial ablation, Mitochondria, Mitochondrial membrane potential, Morphological changes, Apoptosis

## Abstract

**Background:**

As an emerging myocardial ablation technique, the mechanism of nanosecond pulse electric field (nsPEF) ablation is currently less studied. Mitochondria are one of the important membrane structure organelles in cells, participating in numerous life activities within the cell. This study aimed to explore the morphological changes of mitochondria in living cells following nsPEF treatment.

**Methods:**

Myocardial cells were treated with a self-made solid-state LTD high-voltage nanosecond pulse generator with a pulse width of 100 ns for 80 times. The changes in mitochondrial membrane potential and cell apoptosis in rat myocardial cells after nsPEFs were investigated using JC-1 assay kit, apoptosis double staining assay kit, and mitochondrial fluorescence probe.

**Results:**

The results showed that after nsPEF treatment, the mitochondrial membrane potential decreased, apoptosis increased, and the average mitochondrial area decreased from 0.48 µm^2^ in live myocardial cells to 0.16 µm^2^. The average circumference ranges from 3.17 µm dropped to 1.60 µm. The shape factor decreased from 1.92 to 1.41. The aspect ratio has decreased from 2.16 to 1.59. nsPEF treatment induces changes in the morphology of myocardial cell mitochondria.

**Conclusions:**

Based on the results of mitochondrial membrane potential and apoptosis, it can be inferred that under this equipment and parameter conditions, nsPEF treatment first causes changes in mitochondrial morphology, and then initiates the mitochondrial apoptosis pathway, which may provide experimental basis for investigating the potential mechanism of nsPEF ablation of myocardial cells.

**Graphical Abstract:**

**Figure:**
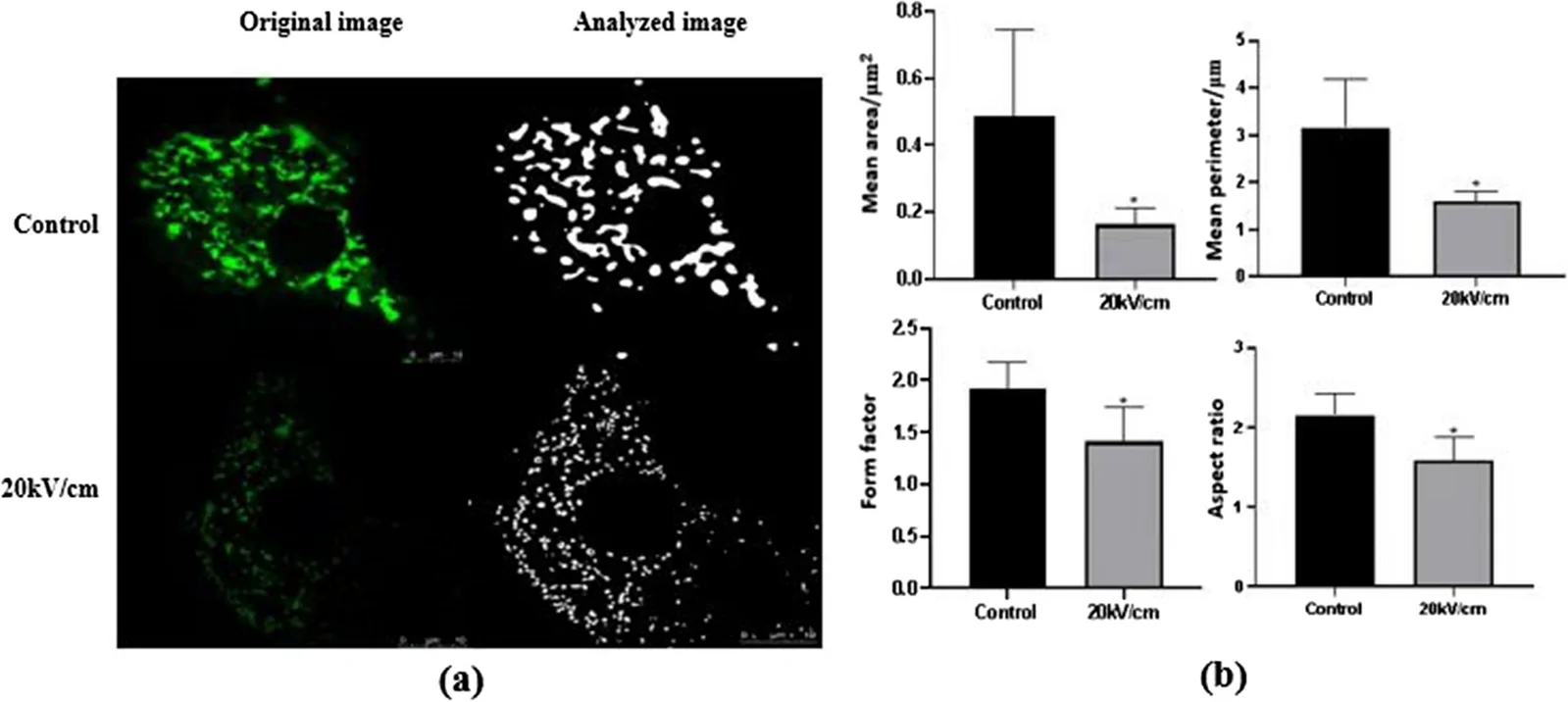

## Introduction

Atrial fibrillation is a condition in which the frequency or rhythm of the heart beat is abnormal due to a disorder in the origin or conduction of the heart’s activity [1, 2]. Mild atrial fibrillation such as sinus arrhythmia usually performs unconspicuous clinical manifestations; however, severe arrhythmia manifested as cardiac arrest, which can be contributed to sudden cardiac death (SCD) [1, 2]. As one of the major death causes all over the world, SCD presents a relatively low survival rate even in hospitalized patients, emphasizing the lack of risk prediction and prevention of atrial fibrillation [3]. Ablation of cardiac tissue is one of the most important tools in the treatment of cardiac arrhythmias, especially in the treatment of atrial fibrillation, atrial flutter, and ventricular tachycardia [4]. Cardiac radiofrequency ablation (RFA) is generally well accepted by doctors and patients for the treatment of arrhythmia [5]. However, certain shortcomings exist, such as high recurrence rate, possible necrosis of tissue cells in non-target tissues, and serious complications such as stroke and esophageal fistula due to the long duration of the ablation procedure as well as thermal side effects including thrombosis [6–8]. Hong et al. [9] performed ablation experiments with microsecond pulses in living sheep hearts and demonstrated that microsecond pulses ablate myocardial tissue; in addition, Stewart et al. [10] and Livia et al. [11] proved the ablation of cardiomyocytes by microsecond pulses using the pulmonary vein of the porcine heart and the dog heart, respectively. There are certain inadequacies, microsecond pulses may perforate cardiomyocyte cells, leading to leakage of cytosol and causing an inflammatory response in the body, and uneven ablation may also occur [6, 12].

As an emerging ablation technology, nanosecond pulse electric fields (nsPEFs) possess better control of ablation volume, shorter process time, and better reduction of thermal side effects [4]. nsPEFs can eradicate the targeted area without causing any thermal harm to surrounding tissues and blood vessels [4]. Current application researches on nsPEFs in myocardial ablation have been primarily centered on identifying the impacts of myocardial ablation at the tissue level and defibrillation at the cellular level [10]. Hargrave et al. [13] discovered that nanosecond pulse treatment improved the enhancement of left ventricular pumping in rabbits, and Xie et al. [4] found that nsPEFs resulted in myocardial ablation with fewer side effects than radiofrequency ablation and microsecond ablation using New Zealand rabbits as the experimental animal.

At present, significant research has yet to be conducted on the morphological changes in mitochondria within live myocardial cells following treatment with nsPEFs. The experiment is dedicated to investigate the morphological changes of mitochondria in living cells following nsPEF treatment using confocal microscopy. This study seeks to comprehensively examine and analyze the effects of nsPEF treatment on the mitochondrial morphology of rat cardiomyocytes and to offer a valuable reference for further research on the mechanism of cardiomyocyte ablation by nsPEFs.

## Materials and methods

### Equipment

The nsPEF equipment utilized in the experiment is an all-solid-state LTD high-voltage nanosecond pulse generator fabricated in the laboratory. It has a pulse width of 100 ns and utilizes the high-voltage probe of Tektronix P5100A connected to a high-speed oscilloscope TDS3054 for real-time monitoring of the pulse voltage values and changes.

### Cell culture and treatment

The rat myocardial cell (H9C2) was obtained from Haixing Biosciences. H9C2 cells were cultured in high-glucose Dulbecco’s modified Eagle’s medium (H-DMEM; Pricella, China) supplemented with 10% fetal bovine serum (FBS; Hangzhou Sijiqing Biological Engineering Co., China) under a humidified atmosphere of 5% CO_2_ and 95% air at 37 °C until the cells covered approximately 80% of the entire bottom of the dish.

**Fig. 1:**
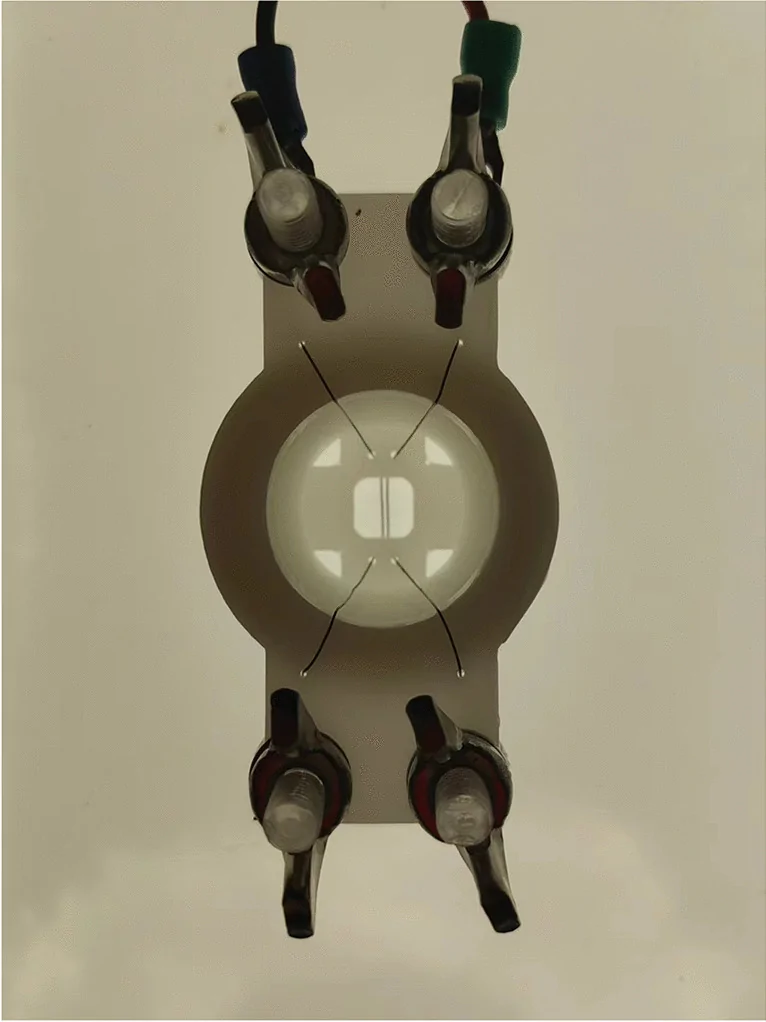
Self-made wall-mounted electrodes with a spacing of 2 mm

The electrode cups processed the cells in the CCK-8 and apoptosis double staining experiments, while the homemade wall-mounted electrodes with a 2-mm spacing were used for the JC-1 and mitochondrial fluorescent probe staining observation experiments. The electrodes were directly affixed to the glass bottom surface of the confocal petri dish, as shown in Fig. 1.

### CCK-8 assay

The CCK-8 assay was performed to assess the effect of nsPEFs on the H9C2 cells according to the instructions (Beyotime, Jiangsu, China). H9C2 cells were transformed into a single-cell suspension using trypsin solution (Pricella, China). The cells were then quantified using a hemocytometer and diluted to a concentration of 2 × 10^6^ cells/mL using DMEM culture medium. Subsequently, 500 µL of the solution was extracted and added to the electrode cups. Nanosecond pulsed electric fields with various field strengths (0 kV/cm, 10 kV/cm, 20 kV/cm, 40 kV/cm) were then applied, with 80 pulses administered to each electrode cup. At the end of the treatment, the concentration of 5000 cells/well was added to the 96-well plate separately after which the 96-well plate was placed in the cell culture incubator. After culture for 24 h, 10 µL of CCK-8 reagent (Beyotime, China) was added to each well with liquid, mixed well, and then put into the cell culture incubator for 30 min. The relative cells were determined by normalizing the optical density (OD) value of the nsPEF-treated group.

### Cell apoptosis assay

Cell apoptosis was measured using the apoptosis detector on the public platform of the Institute of Medical Technology. Cells were subjected to apoptosis induction according to the experimental protocol, and nanosecond pulsed electric fields with different field strengths were applied respectively: 0 kV/cm, 20 kV/cm, and 80 pulses were applied to each electrode cup, and the cells were washed three times with PBS immediately after the treatment, and 5 µL of Annexin V-FITC and propidium iodide (PI) (Beyotime, China) were added in order, and the cells were determined immediately after staining.

### JC-1 detection

For H9C2 cells that had been adhered to the confocal petri dish, JC-1 reagent (Beyotime, China) staining was carried out according to the instructions, and then the cells were treated according to the experimental protocol, respectively, applying nsPEFs with different field strengths: 0 kV/cm and 20 kV/cm, and 80 pulses were applied to each electrode cup, then the cells were observed under a laser confocal microscope at the end of the treatment, and the results were photographed and saved. The results were exported on DVD, and the exported results were analyzed by ImageJ software to obtain the intensity of red fluorescence and green fluorescence, respectively.

### Mitochondrial fluorescence probe assay

The H9C2 cells attached to confocal petri dishes were stained by mitochondrial green fluorescent probe (Beyotime, China) according to the manufacturer’s instructions, with the mitochondrial probe concentration of 100 mm. Cells were performed according to the experimental protocol, and nsPEFs with different field strengths were applied: 0 kV/cm, 20 kV/cm, and 80 pulses were applied to each electrode cup. The cells were observed under a laser confocal microscope at the end of the treatment; the results were photographed, saved, and exported on DVD. Then, the results were analyzed with ImageJ software to obtain the changes in the morphology and number of the mitochondria.

### Statistical analysis

The data were statistically analyzed using GraphPad Prism 9. All samples were assayed simultaneously on the same plate to avoid operational variations. Each sample was assayed at least 3 times and the mean concentration was calculated. One-way ANOVA was used to ascertain statistically significant variances (*P* < 0.05).

## Results

### Effect of nsPEFs on the viability of H9C2 cells

**Fig. 2:**
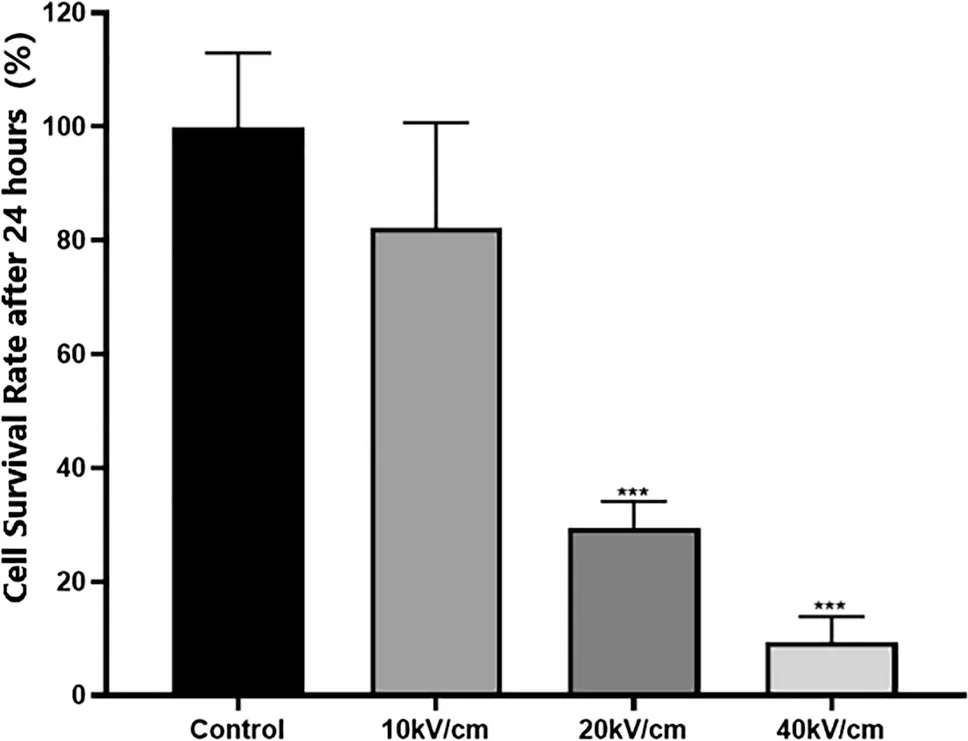
The variation of cell survival rate with field strength. The horizontal axis represents the applied field strength of the nanosecond pulse electric fields used in the experiment, the vertical axis represents the cell survival rate after 24 h of nanosecond pulse electric field treatment, and control represents the field strength of 0 kV/cm after nanosecond pulse electric field treatment. The values in the columns in the figure represent the specific values of cell survival rate relative to the control group. *** represents *P* < 0.05

The CCK-8 assay was determined to evaluate the viability of H9C2 cells at the nsPEF strengths of 0 kV/cm, 10 kV/cm, 20 kV/cm, and 40 kV/cm. According to Fig. 2, it can be seen that with the increase of the nsPEF strength, the cell survival rate decreased significantly when the electric field strength was 20 kV/cm and above, at 20 kV/cm, the cell survival rate was only 29% compared to the control group, and at 40 kV/cm, the cell survival rate was only 9%. The survival rate was extremely low, not applicable to the late experiments; therefore, the selected parameters 20 kV/cm and 80 pulses were applied in the nsPEF group after this experiment.

### Effect of nsPEFs on the apoptosis of H9C2 cells

**Fig. 3:**
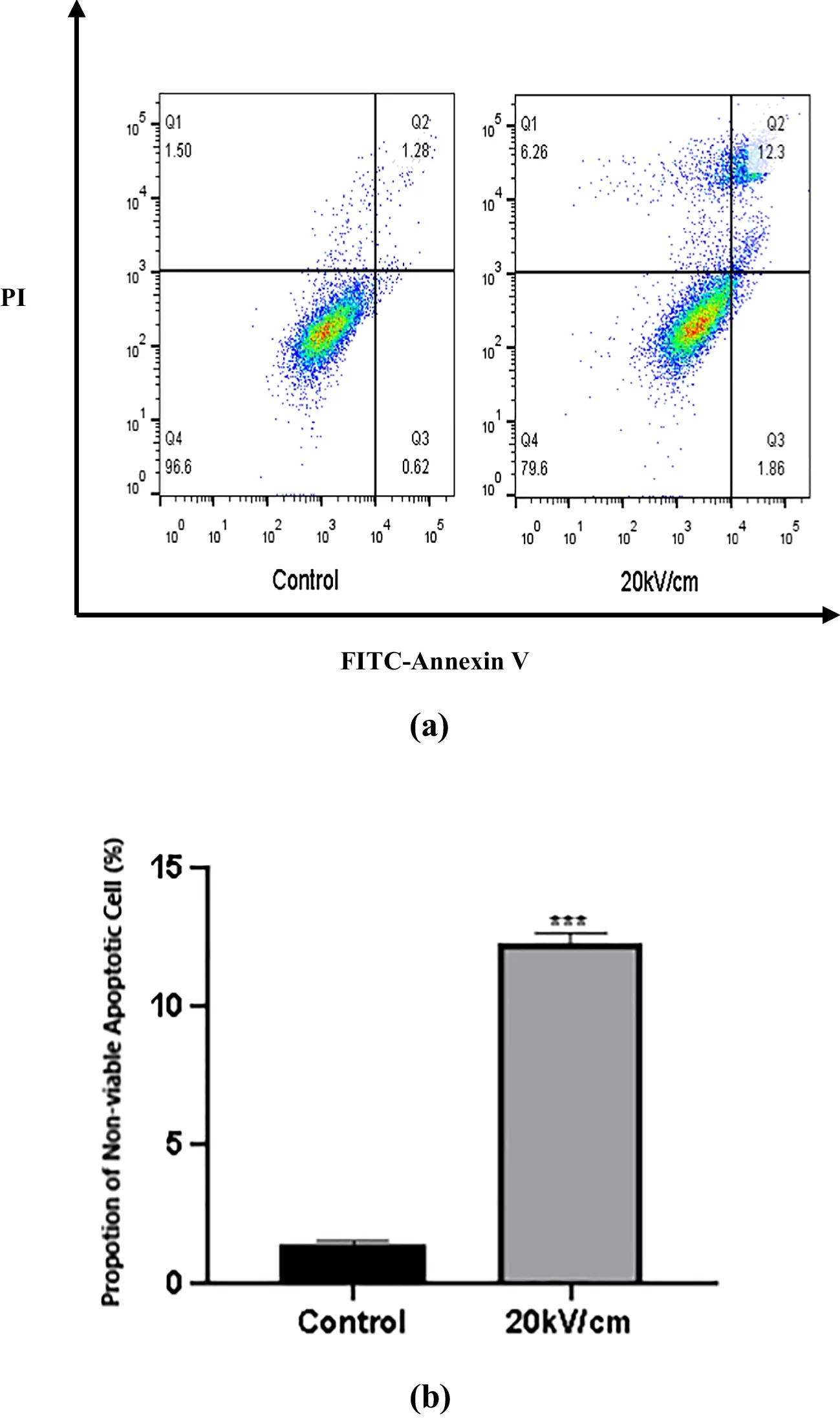
Flow cytometry results of rat cardiomyocytes treated with nanosecond pulsed electric fields for 2 h. **a** The results of flow cytometry detection of apoptotic cells, with FITC channels used in the horizontal axis and PI channels used in the vertical axis. The field strength in each small image is the nanosecond pulse electric field strength used in the experiment. Each small image is divided into four regions, each representing a different cell state: Q1 in the upper left corner represents mechanical injury, Q2 in the upper right corner represents late cell apoptosis or necrosis, Q3 in the lower left corner represents normal cells, and Q4 in the lower right corner represents early cell apoptosis. The value of each region is the ratio of the cells represented by each region to the total cells, which is the result of omitting the percentage sign. **b** The results of Prism9 statistical analysis. *** represents *P* < 0.001

Flow cytometry was performed to explore the effect of nsPEFs on the apoptosis of H9C2 cells, the results showed that the proportion of apoptotic cells in the untreated group was 1.67% of the total cells, and the proportion of late apoptotic and necrotic cells in the group with the field strength of 20 kV/cm was 12.28% of the total cells, which was extremely significant compared with that in the untreated group (Fig. 3).

**Fig. 4:**
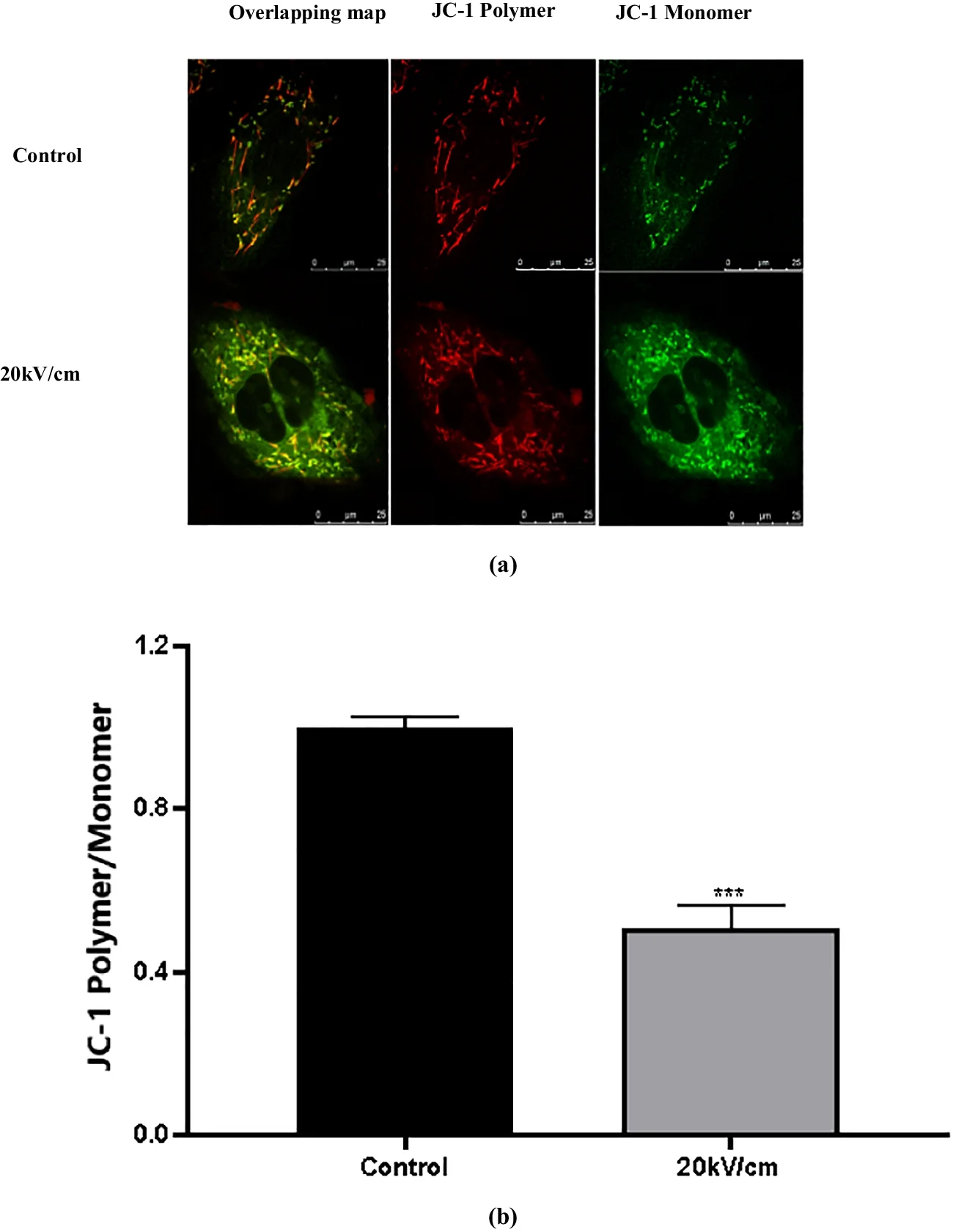
JC-1 result graph after nanosecond pulse electric field treatment. **a** The results of the nanosecond pulse electric field strengths captured by a laser confocal microscope at 0 kV/cm and 20 kV/cm, respectively. The control group is 0 kV/cm, and the selected parameters, such as voltage and noise reduction, are consistent for both groups when captured on the laser confocal microscope. **b** The summary of ImageJ’s analysis of the results in **a**, which were then analyzed using Prism9 software. The horizontal axis represents the applied field strength of the nanosecond pulse electric fields used in the experiment, the vertical axis represents the cell survival rate after 24 h of nanosecond pulse electric field treatment, and control represents the field strength of the nanosecond pulse electric fields as 0 kV/cm. The values in the columns in the figure represent the specific values of cell survival rate relative to the control group. *** represents *P* < 0.001

The mitochondrial membrane potential test kit (JC-1) was used to detect the status of mitochondria, as shown in Fig. 4, the ratio of JC-1 monomer/polymer was decreased from 1 to 0.51, and the ratio of JC-1 monomer/polymer was directly reflecting the change of the mitochondrial membrane potential. Moreover, the change of the mitochondrial membrane potential was basically the same as that of the results of the apoptotic double staining assay. When the field strength of nsPEFs was 20 kV/cm, the mitochondrial membrane potential in the cell was significantly reduced, and the cell survival rate was also significantly reduced.

### Changes of mitochondrial morphology in H9C2 after nsPEF treatment

**Fig. 5:**
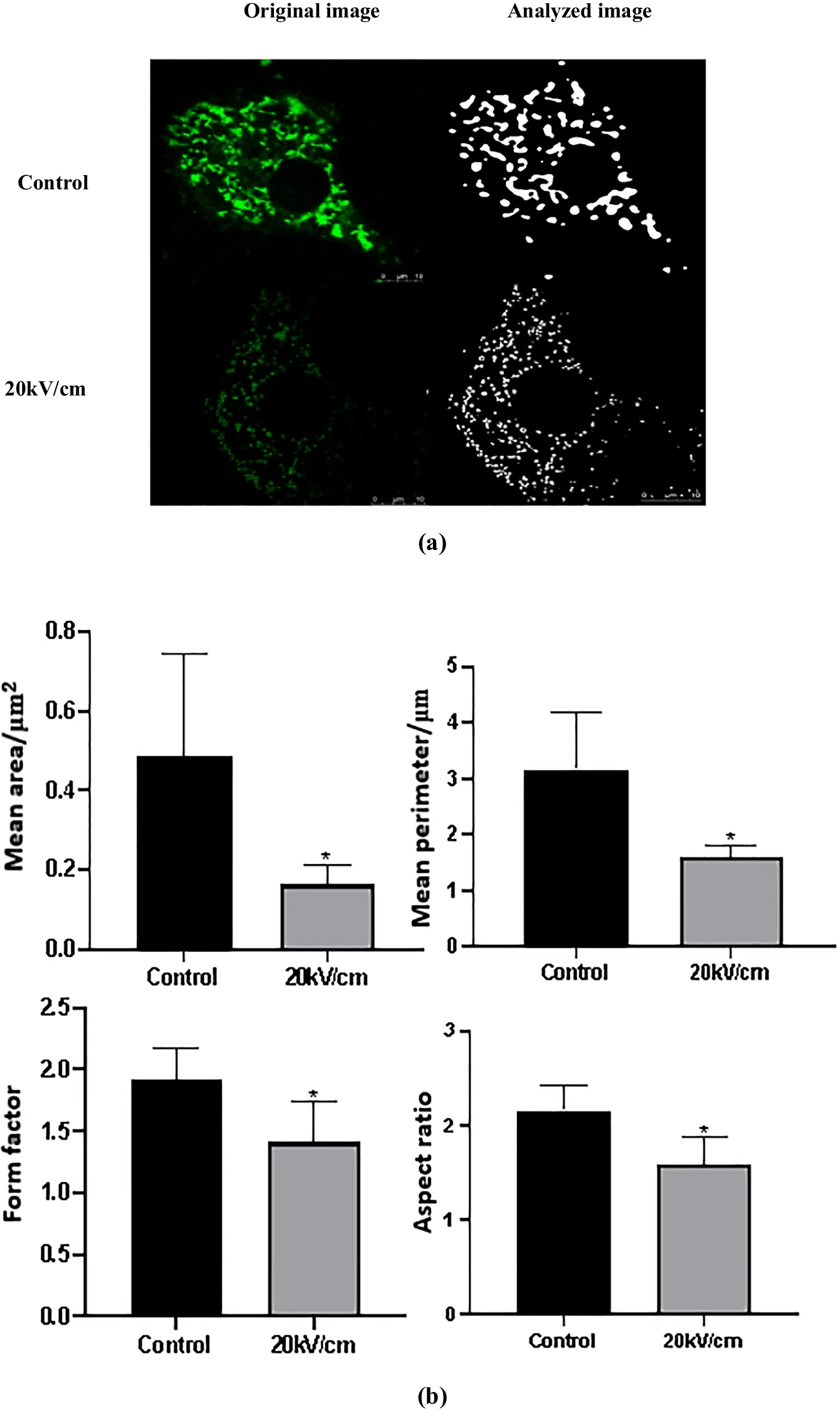
Results of mitochondrial morphological changes in myocardial cells after nanosecond pulsed electric field treatment. **a** The results of the nanosecond pulse electric field strengths captured by a laser confocal microscope at 0 kV/cm and 20 kV/cm, respectively. The control group is in the 0 kV/cm group, with the original image captured on the left and the ImageJ analysis image on the right. The parameters selected for shooting the two sets of result images on a laser confocal microscope, such as voltage and noise reduction, remain consistent. **b** The summary of ImageJ’s analysis of the results in **a**, which were then analyzed using Prism9 software. The horizontal axis represents the applied field strength of the nanosecond pulse electric fields used in the experiment, the vertical axis represents the mitochondrial changes after nanosecond pulse electric field treatment, and control represents the nanosecond pulse electric field strength of 0 kV/cm. The values in the columns in the figure represent the specific values of cell survival rate relative to the control group. * represents *P* < 0.05

The results of mitochondrial fluorescence probe experiment were captured by laser confocal microscopy and analyzed by ImageJ software, which showed that nanosecond pulsed electric field treatment led to significant changes in the number of mitochondrial morphologies in living myocardial cells, resulting in significant decreases in the mean area, mean perimeter, form factor, and aspect ratio of mitochondria, with the mean area decreasing from 0.48 to 0.16 µm^2^; the mean perimeter decreasing from 3.17 to 1.60 µm; the form factor decreasing from 1.92 to 1.41; and the aspect ratio decreasing from 2.16 to 1.59; average perimeter decreased from 3.17 to 1.60 µm; shape factor decreased from 1.92 to 1.41; and aspect ratio decreased from 2.16 to 1.59 (Fig. 5).

## Discussion

nsPEFs is known as a burgeoning technology in cardiac ablation and has proved to be effective in ablating cardiac cells with fewer side effects than RFA [14]. In this study, we found a dose–effect relationship between nsPEF field strength and rat cardiomyocyte survival, with higher field strengths resulting in lower survival. nsPEF treatment of rat cardiomyocytes reduces cell viability mainly by inducing apoptosis. In addition, nsPEF treatment leads to significant changes in mitochondrial morphology, initiating the mitochondrial apoptotic pathway.

At present, the research on the application of nsPEFs in myocardial ablation mainly focuses on the detection of the effect of myocardial ablation at the tissue level and the defibrillation effect at the cellular level. However, at the cellular level, studies on the apoptosis of cardiomyocytes induced by nanosecond pulsed electric field and the mechanism of its induced ablation of myocardial tissues are still insufficient, and the morphological changes of mitochondria in myocardial living cells after nanosecond pulsed electric field have not yet been observed. In this experiment, we investigated the morphological changes of mitochondria in living rat myocardial cells with the change of the field strength of nanosecond pulsed electric field by using JC-1 stain, apoptosis double staining reagent, and mitochondrial fluorescent probe.

Previous studies have shown that nanosecond pulses can cross the cell membrane and cause irreversible electroporation of mitochondria by altering the transmembrane potential of mitochondria, ultimately triggering apoptosis of tumor cells, which was subsequently confirmed by a great deal of researches [15–18]. Weaver et al. [19] suggested that the width of the nanosecond pulse is less than the charging time of the cell membrane, and that the nanosecond pulse can penetrate the cell and act on organelle structures before the cell membrane is fully charged. Electroporation is induced in the nucleus and mitochondria, and intracellular calcium ions are released, leading to apoptosis. With the development of nsPEF technology, abundant investigations of cellular and animal experiments on the biological effects of nanosecond pulses have been carried out in the biomedical field [20]. nsPEF shows a promising application in the field of myocardial ablation due to its low side effects [21]. However, most of the current studies in the field of nsPEF-cardiac ablation have been conducted directly on animal models, and very few in-depth studies focus on the specific mechanism of ablation [9–11, 22]. The mitochondrial apoptotic pathway, as one of the apoptotic pathways, is a noteworthy direction to explore the mechanism of myocardial ablation by nsPEFs.

Mitochondrial fluorescent probe was used to detect the morphological changes in rat cardiomyocytes after nsPEFs, and the mitochondrial fluorescent probe results displayed that nsPEF treatment led to the significant reduction of mitochondrial mean area, mean perimeter, shape factor, and aspect ratio. nsPEF treatment results in “electroporation” phenomenon; combined with the previous study of Ma et al. [23], it can be speculated the reasons for the changes in the morphology of the mitochondria: first, nsPEF treatment will make the mitochondrial membrane “electroporation” phenomenon and the mitochondrial membrane “electroporation” seriously leads to mitochondrial fracture under the conditions of this equipment and parameters; second, nsPEF treatment is involved in the mitochondrial fusion and division process, which promotes the mitochondrial division. Combined with the results of JC-1 experiments, flow results, and cell viability results, the first hypothesis is more likely. Corovic et al. [24] explored the distribution of pulsed electric fields around the electrodes and showed that the extent of ablation of cells subjected to pulsed electric fields correlates with the extent of the electric field coverage, and that only cells in the tissue exposed to a sufficiently high electric field become permeable and the cell membranes become electroporated. The study of the mechanism of ablation of cardiomyocytes by nsPEFs is currently limited to animal experiments, and further studies are needed in the future to explore the wide application of nsPEFs.

## Conclusion

Combining the results of previous experiments with the results of JC-1 experiments, flow results, and cell viability results in this paper, the hypothesis can be obtained that under the conditions of this equipment and parameters, nsPEFs initiate the mitochondrial apoptosis pathway by inducing changes in mitochondrial morphology.

## Data Availability

The datasets analyzed in this study are available from the corresponding author upon reasonable request.
